# Diversity of Sulfur-oxidizing Bacteria at the Surface of Cattle Manure Composting Assessed by an Analysis of the Sulfur Oxidation Gene *soxB*

**DOI:** 10.1264/jsme2.ME18066

**Published:** 2020-07-22

**Authors:** Yumi Mori, Chika Tada, Yasuhiro Fukuda, Yutaka Nakai

**Affiliations:** 1 Laboratory of Sustainable Animal Environmental Science, Graduate School of Agricultural Science, Tohoku University, 232–3 Yomogida, Naruko-onsen, Osaki, Miyagi 989–6711, Japan; 2 Research Institute for Bioresource and Biotechnology, Ishikawa Prefectural University, 1–308 Suematsu, Nonoichi, Ishikawa 921–8836, Japan

**Keywords:** cattle manure compost, cloning analysis, *Proteobacteria*, *soxB*, sulfur-oxidizing bacteria

## Abstract

Sulfur-oxidizing bacterial diversity at the surface of cattle manure was characterized throughout the composting process using a sulfur oxidation gene (*soxB*) clone library approach. In the mesophilic phase, clones related to the genera *Hydrogenophaga* and *Hydrogenophilus* were characteristically detected. In the thermophilic phase, clones related to the genera *Hydrogenophaga* and *Thiohalobacter* were predominant. In the cooling phase, the predominant *soxB* sequences were related to the genus *Pseudaminobacter* and a new sulfur-oxidizing bacterium belonging to the class *Alphaproteobacteria*. The present study showed changes in the community composition of sulfur-oxidizing bacteria at the surface of compost throughout the composting process.

Chemolithotrophic sulfur-oxidizing bacteria (SOB) are aerobic bacteria that belong to the phylum *Proteobacteria* and oxidize reduced inorganic sulfur compounds to sulfate (Friedrich, 1997), thereby contributing to the sulfur cycle. The role of SOB in various environments has been investigated using polymerase chain reaction (PCR) primers targeting the 16S rRNA gene and the functional genes *soxB*, *apr*, and *dsrAB*, which are involved in sulfur-oxidation biochemical pathways (Meyer and Kuever, 2007; Hügler *et al.*, 2010; Luo *et al.*, 2011; Tourova *et al.*, 2012; Headd and Engel, 2013; Mori *et al.*, 2013; Kojima *et al.*, 2014; Xia *et al.*, 2014).

Composting is an exothermic process involving the biodegradation of organic waste materials, such as animal manure. The composting process comprises three phases (Keener *et al.*, 2000): (i) a mesophilic phase lasting one to three days, during which mesophilic bacteria and fungi degrade organic matter, such as sugars, amino acids, and proteins, and the compost temperature rapidly increases; (ii) a thermophilic phase lasting 10 to as many as 100 days, during which the compost temperature increases to higher than 60°C, and fats, hemicellulose, cellulose, and some lignins are degraded; and (iii) a cooling phase lasting 10 to 100 days that is characterized by a decrease in the compost temperature due to a reduction in microbial activity. SOB have been detected in manure compost, and may contribute to preventing the evaporation of hydrogen sulfide from compost via sulfide oxidization (Beffa *et al.*, 1995, 1996; Asano *et al.*, 2007). In addition, sulfate produced by SOB reduces the pH of compost, thereby decreasing the volatility of ammonia and its subsequent evaporation (Stevenson, 1985; Mari *et al.*, 2005; Gu *et al.*, 2011). Since the evaporation of ammonia from compost causes odor, acid rain, and reduced fertilizer quality, SOB may contribute to the alleviation of odor, reductions in environmental pollutants, and maintenance of fertilizer quality. However, temporal changes in the composition of the SOB community throughout the composting process remain unknown.

The SoxB component of the periplasmic thiosulfate-oxidizing Sox enzyme complex is regarded as a primordial, but fundamental, molecular mechanism for sulfur oxidation (Friedrich *et al.*, 2001; Meyer *et al.*, 2007). The Sox enzyme complex is proposed to be widespread among various phylogenetic groups of SOB (Friedrich *et al.*, 2001, 2005; Hensen *et al.*, 2006). The findings of a phylogenetic analysis of *soxB* and physiologically divergent SOB strains were largely congruent with the 16S rRNA gene-based phylogenetic lineage (Meyer *et al.*, 2007). Tourna *et al.* (2014) recently developed a molecular approach to specifically identify chemolithotrophic SOB in soil ecosystems using primers specifically targeting *soxB*, the functional gene of SOB, for the classes *Alphaproteobacteria*, *Betaproteobacteria*, and *Gammaproteobacteria*. Since the physical and chemical parameters of compost may markedly change within the time span of a few days, it is important to investigate SOB ecology during all phases of the composting process in order to reduce odor and produce high-quality compost. In the present study, we investigated the diversity of *soxB* at the surface of cattle manure throughout the composting process.

The temperature at the start of the composting process was approximately 45°C, and in less than two days, increased to approximately 60°C. After mixing, the compost temperature decreased and then increased again (Fig. S1). After day 23, the compost temperature remained lower than 60°C and decreased to 42°C by day 106 (Fig. S1). Based on these results, the mesophilic, thermophilic, and cooling phases of composting were defined as days 0–2, days 3–22, and days 23–106, respectively. We obtained samples for chemical and molecular analyses from three distinct locations within the compost pile on five specific days of the composting process, which represented the mesophilic phase (day 1), thermophilic phase (day 4), and cooling phase (days 38, 64, and 106). The concentration of ammonium increased from 0.71 to 1.23‍ ‍mg N [g dry compost]^–1^ from the mesophilic phase to the thermophilic phase, and decreased to 0.04‍ ‍mg N [g dry compost]^–1^ throughout the cooling phase (Table 1). The concentration of nitrate increased from 0.02 to 1.04‍ ‍mg N [g dry compost]^–1^ from the thermophilic phase to the end of the cooling phase (Table 1). The profiles of ammonium and nitrate concentrations indicated that organic nitrogen was decomposed to ammonium during the mesophilic phase to the thermophilic phase, while ammonium was oxidized to nitrate during the cooling phase (Table 1). The concentration of sulfate increased throughout the composting process. The accumulation of sulfate indicated that reduced sulfur compounds were oxidized to sulfate by SOB throughout the composting process (Table 1). Its accumulation also reduced pH during the composting process (r^2^=–0.89). This result indicated that SOB contribute to preventing the evaporation of hydrogen sulfide and ammonia throughout the composting process.

We detected phylogenetically diverse *soxB* genes in all the samples analyzed by PCR and subsequent cloning procedures. Detailed procedures are described in the Supplementary Methods. A total of 260 *soxB* clones were sorted into 30 operational taxonomic units (OTUs) based on our parameter of >80% sequence identity (Table S1) (Headd and Engel, 2013). The clones in OTU1–OTU8, OTU13–OTU14, and OTU9–OTU12 and OTU15–OTU30 belonged to the classes *Betaproteobacteria*, *Gammaproteobacteria*, and *Alphaproteobacteria*, respectively (Fig. 1). The SOB community composition exhibited changes throughout the composting process. During the mesophilic phase, bacteria belonging to the classes *Alphaproteobacteria* and *Betaproteobacteria* accounted for 53 and 47% of the SOB community, respectively, while during the thermophilic phase, SOB belonging to the classes *Alphaproteobacteria*, *Betaproteobacteria*, and *Gammaproteobacteria* accounted for 26, 28, and 46%, respectively (Fig. S2). During the cooling phase, SOB belonging to the class *Alphaproteobacteria* dominated and comprised 69–90% in each clone library (Fig. S2).

During the mesophilic phase, OTU1 and OTU4 clones, belonging to the class *Betaproteobacteria*, accounted for 20.4 and 18.4%, respectively (Table S1). These clones were the primary SOB and may be major contributors to sulfur oxidization during the mesophilic phase. OTU1 clones were phylogenetically related to *Hydrogenophilus thermoluteolus* (Fig. 1), which uses thiosulfate as an electron donor and was originally isolated from soil around a hot spring (Hayashi *et al.*, 1999; Miyake *et al.*, 2007). Sequence identity between the OTU1 clone and *H. thermoluteolus* was 70.7%. The optimum temperature and pH of *H. thermoluteolus* are reported to be 50–52°C and approximately 7.0 (Hayashi *et al.*, 1999), respectively, differing from the conditions of the mesophilic phase in the present study, which were 45°C and 9.47, respectively (Table 1). Therefore, OTU1 clones may represent new SOB related to the genus *Hydrogenophilus*, with different physiological properties from those of *H. thermoluteolus*. OTU4 clones formed a clade with the SOB of the genus *Hydrogenophaga* (Fig. 1), which was isolated from compost (Beffa *et al.*, 1996). SOB comprising OTU4 may share many of the same physiological properties with the genus *Hydrogenophaga* because sequence identity between the OTU4 clone and *Hydrogenophaga* sp. B11 was 91.5%. Specific *Hydrogenophaga* species are capable of thiosulfate oxidation (Graff and Stubner, 2003), and, thus, OTU4 clones may oxidize reduced sulfur compounds, such as sulfide and thiosulfate, during the mesophilic phase. On the other hand, the genera *Hydrogenophaga* and *Hydrogenophilus* oxidize hydrogen (Hayashi *et al.*, 1999; Willems and Gillis, 2005). Hydrogen is produced by H_2_-producing bacteria from anaerobically fermented cow waste slurry (Yokoyama *et al.*, 2007; Guo *et al.*, 2010). Since the deep regions of compost and the inside of aggregated manure structures become anaerobic, OTU1 and OTU4 clones may oxidize the hydrogen generated in the anaerobic environments within compost.

In the thermophilic phase, the diversity of SOB was the lowest throughout the composting process (Table S2). OTU4 clones belonging to the class *Betaproteobacteria* and OTU14 clones of the class *Gammaproteobacteria* were the main SOB, accounting for 27.9 and 44.3%, respectively (Table. S1).

OTU4 clones belonging to the genus *Hydrogenophaga* were also predominant during the thermophilic phase (Fig. 1, Table S1). Since sulfate accumulated from days 1–4, OTU4 clones may have oxidized reduced sulfur compounds. On the other hand, the hydrogen-oxidizing bacterial genus *Hydrogenophaga* has been detected in cattle manure compost that was anaerobically fermented at 75°C (Yokoyama *et al.*, 2007). In manure compost, the deeper areas become anaerobic, and temperature increases to >70°C, particularly at the thermophilic phase (Miyatake and Iwabuchi, 2005). Although the surface temperature of compost was 58°C in the present study, that inside compost may have been higher than 70°C. The amount of hydrogen generated from anaerobically fermented cattle manure at 75°C was previously reported to be 25-fold higher than that at 37°C (Guo *et al.*, 2010). In the present study, high concentrations of hydrogen may have been generated from deeper areas of compost at the thermophilic phase. Therefore, the OTU4 clone may also have oxidized the hydrogen produced in the deep part of compost; as a result, the OTU4 clone dominated during the thermophilic phase.

OTU14 clones were found to be phylogenetically related to the halophilic SOB *Thiohalobacter thiocyanaticus*, which was isolated from hypersaline lakes (Fig. 1) (Sorokin *et al.*, 2010). They may contribute to sulfur oxidization during the thermophilic phase. The most closely related nucleotide sequence is *Hydrogenophaga* sp. RAC07; however, in the phylogenetic tree based on amino acid sequences, *T. thiocyanaticus* was found to be the most closely related to OTU14 clones (Fig. 1 and Table S1). Since amino acid sequences reflect function rather than nucleotide sequence information, we discussed physiological similarities between OTU14 and *T. thiocyanaticus* in the present study. Sequence identity between the OTU14 clone and *T. thiocyanaticus* was only 78.1%. Furthermore, the optimal growth temperature and pH for *T. thiocyanaticus* (20–40°C and pH 6.3–8.0) were both lower than those observed in the present study on day 4 (58°C and pH 9.45). Therefore, OTU14 clones may be new SOB with different physiological properties from those of *T. thiocyanaticus*. By isolating SOB belonging to OTU14 and elucidating their physiological characteristics, we may be able to establish why OTU14 dominated in the thermophilic phase.

During the cooling phase, clones belonging to the class‍ ‍*Alphaproteobacteria* comprised 69% or more of each library (Fig. S2). The clones OTU9, OTU20, OTU24, OTU26, and OTU27 were predominant (Table S1). These *Alphaproteobacteria* clones may contribute to the oxidation of reduced sulfur compounds during the cooling phase. OTU20 clones were phylogenetically related to sulfur-oxidizing *Pseudaminobacter salicylatoxidans* (Fig. 1), which is detected in soil (Deb *et al.*, 2004). Calculated sulfur accumulation rates were 0.0100, 0.0050, 0.0004, and 0.0293‍ ‍mg S g^–1^ d^–1^ from days 1–4, days 4–38, days 38–64, and days 64–106, respectively. At the end of the cooling phase (from days 64–106), the sulfate accumulation rate was the highest. Since OTU20 clones were dominant (33.3%) on day 106, they may have contributed to the high activity of sulfur oxidation at the end of the cooling phase. Sequence identity between the OTU20 clone and *P. salicylatoxidans* was not high (77.0%), and the growth temperature range of *P. salicylatoxidans* (20–40°C) differed from that measured during the cooling phase (41–52°C) (Kämpfer *et al.*, 1999). Therefore, clones of OTU20 may be new SOB that have adapted to higher temperatures than *P. salicylatoxidans*. *P. salicylatoxidans* and *Mesorhizobium* sp. B7, which were also phylogenetically related to OTU20 clones (Fig. 1), have the ring-fission dioxygenase, which confers these organisms with the ability to cleave carbon rings (Kämpfer *et al.*, 1999; Eppinger *et al.*, 2016; NCBI Reference Sequence NZ_CP018171.1). Fumic acid, an aromatic compound containing carbon rings, is present in cattle manure compost and accumulates throughout the composting process (Bernal *et al.*, 2009). The increased detection frequency of clone OTU20 after day 38 indicates that this clone possesses the ring-fission dioxygenase and degrades stable organic matter, such as fumic acid. Clones OTU24, OTU26, and OTU27 were detected after day 38 and comprised 2.5–27.5% of each library (Table S1). These clones were not related to any previously isolated SOB (Fig. 1). Therefore, they may be novel SOB adapted to the environment of the cooling phase. OTU9 clones were detected at a prevalence of 14% or more in all libraries, except for day 106 (Table S1). According to the phylogenic analysis, they were related to *Azospirillum thiophilum* (Fig. 1), which oxidizes thiosulfate and was initially isolated from a sulfide spring (Lavrinenko *et al.*, 2010; Frolov *et al.*, 2013). Based on their presence, OTU9 clones appear to contribute to sulfur oxidation throughout the composting process. *A. thiophilum* grows at 15–40°C and pH 6.5–8.5 under microaerobic conditions (Frolov *et al.*, 2013; Kojima *et al.*, 2014). Temperature and pH from days 1–64 (41–61°C and pH 8.57–9.47) were higher than the conditions reported for *A. thiophilum*. Sequence identity between OTU9 and *A. thiophilum* was only 77.8%. Therefore, OTU9 clones may be new SOB that are adapted for higher temperatures and more alkaline conditions than *A. thiophilum*.

Ammonia volatilization from cattle manure compost primarily occurs during the thermophilic phase (Pagans *et al.*, 2006). The abundance of ammonia and ammonium ions vary with pH. Between pH 7 and 9.25, the abundance of ammonium ions is higher than that of ammonia. At pH higher than 9.25, the abundance of ammonia is higher than that of ammonium ions (Kunz and Mukhtar, 2016). Ammonia may have volatilized from compost throughout the mesophilic and thermophilic phases because pH in compost was higher than 9.25 on day 1 and day 4 (Table 1). After day 38, ammonia volatilization decreased because pH was lower than 9.25 (Table 1). pH decreased from days 4–38, while the concentration of sulfate markedly increased. Therefore, SOB detected on day 4 (SOB related to *T. thiocyanaticus*, SOB belonging to the genus *Hydrogenophaga*) and day 38 (SOB related to *A. thiophilum*, *P. salicylatoxidans*, and new *Alphaproteobacteria*) produced sulfate, lowered pH in compost, and contributed to the suppression of ammonia volatilization.

In conclusion, SOB belonging to the classes *Alphaproteobacteria* and *Betaproteobacteria* appear to be key players in sulfur oxidation during the mesophilic phase. In the thermophilic phase, SOB belonging to the classes *Betaproteobacteria* and *Gammaproteobacteria* were the main sulfur oxidizers. During the cooling phase, SOB from the class *Alphaproteobacteria* may constitute key players in sulfur oxidation. Our cloning analysis also revealed the presence of a new species of SOB that was detected for the first time in the present study. To the best of our knowledge, this is the first molecular study to examine SOB throughout the cattle manure composting process. Although the physiological identities of SOB and the activity of sulfur oxidation in compost have yet to be examined, the present results provide important information and serve as a foundation for further studies.

## Nucleotide sequence accession numbers

*soxB* sequence data have been submitted to the DDBJ database under accession numbers LC210254–LC210513.

## Citation

Mori, Y., Tada, C., Fukuda, Y., and Nakai, Y. (2020) Diversity of Sulfur-oxidizing Bacteria at the Surface of Cattle Manure Composting Assessed by an Analysis of the Sulfur Oxidation Gene *soxB*. *Microbes Environ ***35**: ME18066.

https://doi.org/10.1264/jsme2.ME18066

## Supplementary Material

Supplementary Material

## Figures and Tables

**Fig. 1.**
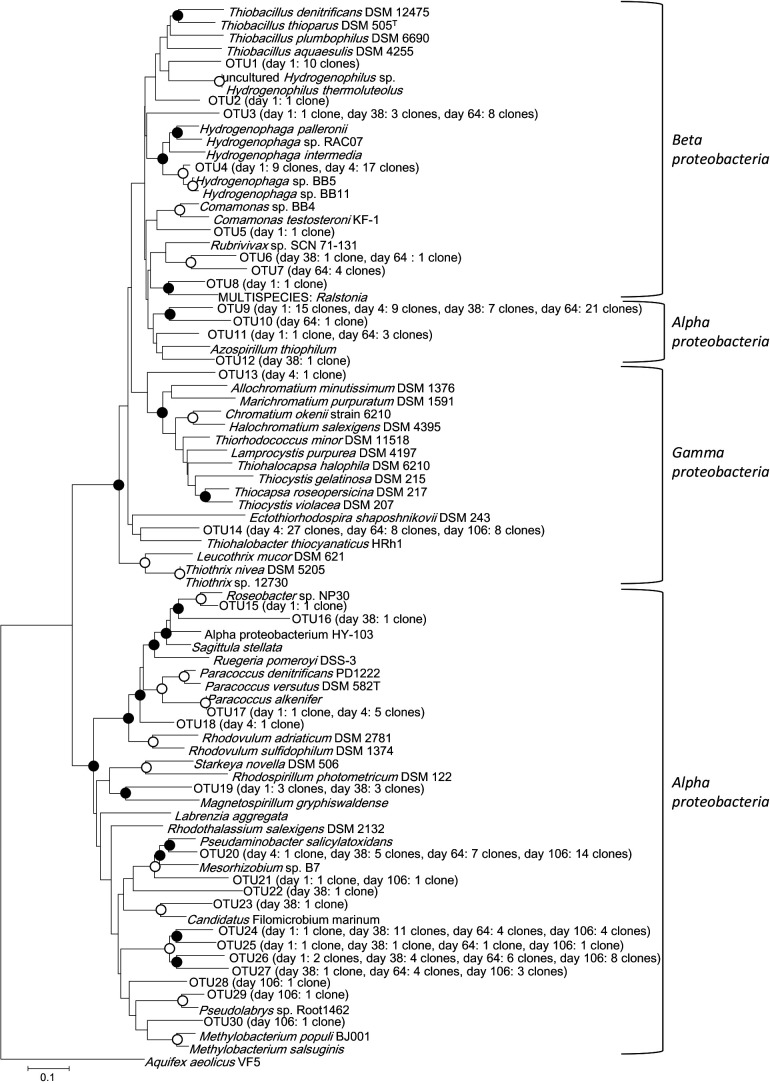
Phylogenetic tree based on translated amino acid sequences of the sulfur oxidation gene *soxB* from clone libraries and selected sequences of described species. The tree was inferred using the minimum evolution method. Values in parentheses are the number of clones within each operational taxonomic unit (OTU). Filled and open circles on the nodes indicate bootstrap values of >50% and >80%, respectively (1,000 replicates).

**Table 1.** Physicochemical properties of compost samples used in the present study

	Temp (°C)	pH	Water content (%)	NH_4_^+^ (mg N [g dry compost]^–1^)	NO_2_^–^ (mg N [g dry compost]^–1^)	NO_3_^–^ (mg N [g dry compost]^–1^)	SO_4_^2–^ (mg S [g dry compost]^–1^)	Cl^–^ (mmol [g dry compost]^–1^)
day 1	45	9.47	62.5	0.71	0.03	0.04	0.10	0.26
day 4	58	9.45	66.2	1.23	0.01	0.02	0.13	0.26
day 38	41	8.68	53.4	0.20	0.01	0.51	0.30	0.33
day 64	52	8.57	50.2	0.17	0.01	0.67	0.31	0.33
day 106	42	7.79	42.6	0.04	not detected	1.04	1.54	0.53
