# Supplementary material for: Diversity of Sulfur-oxidizing Bacteria at the Surface of Cattle Manure Composting Assessed by an Analysis of the Sulfur Oxidation Gene soxB
Source: Microbes Environ. 2020 Jul 22;35(3):ME18066. doi: 10.1264/jsme2.ME18066 (PMC7511791; doi:10.1264/jsme2.ME18066)
Supplement: Supplementary file 1 — Supplementary Material [file 35_18066_s1.pdf]

Supplementary information for "Diversity of Sulfur-oxidizing Bacteria at the Surface of Cattle Manure Assessed by an Analysis of the Sulfur Oxidation Gene *soxB*"

Yumi Mori, Chika Tada, Yasuhiro Fukuda, Yutaka Nakai

Supplementary Methods

Sample collection

Compost samples consisting of cattle feces, straw bedding, sawdust, and recycling compost, were collected from a field-scale facility (approximately 40–50 t) owned by the Graduate School of Agricultural Science, Tohoku University. The day on which the raw materials were mixed was considered as day 0. The combined materials were mixed by rotary mixer every 3 or 4 days until day 36. After day 36, the compost was stirred using a wheel loader approximately every 30 days until day 106. From day 0 to day 36, air was blown upward from the bottom of the compost. Samples for DNA extraction and chemical analyses were collected at a 30 cm depth from three different places on days 1, 4, 38, 64, and 106 following the initiation of the composting process. Three samples derived from the same sampling days were mixed and stored at −80°C until analyzed.

Chemical and physical parameters

Prior to stirring, the temperature was measured with a thermometer at a depth of 30 cm from the surface of the compost. The mixed compost sample was divided into three subsamples and the following treatment was performed. The compost subsamples were dried at 105°C and then weighed to measure the water content. Compost extracts were prepared by adding 3 g of compost to 27 mL of distilled water with stirring for 10 min. The extracts were centrifuged and the water-based supernatant was used to measure the pH (Accumet-AR25; Fisher Scientific, Pittsburgh, PA, USA). The supernatant was filtered using 0.45-μm cellulose acetate filters (Advantec Toyo, Tokyo, Japan) and ammonium, nitrite, nitrate, sulfate, and chloride ion concentrations were measured using the ICS-1000 and ICS-2000 ion chromatography systems (Dionex, Sunnyvale, CA, USA).

DNA extraction

DNA was extracted from 10 g of the compost samples using the PowerMax Soil DNA isolation kit (Mo Bio Laboratories Inc., Carlsbad, CA, USA) according to the manufacturer's instructions. DNA concentrations were quantified using a Nano-Drop 2000 spectrophotometer (Thermo Scientific, Wilmington, DE, USA). Extracted DNA was stored at −20°C.

Cloning and sequencing of soxB

PCR was performed using primers 710F (5′-ATCGGYCAGGCYTTYCCSTA-3′) and 1184R (5′-MAVGTGCCGTTGAARTTGC-3′), which target the *soxB* encoding the SoxB subunit of the Sox enzyme system (7). The reaction mixture (25 μL) consisted of 1 U EX Taq polymerase (Takara Bio, Shiga, Japan), 1× reaction buffer, 0.5 μM of the forward and reverse primers, 0.2 μM dNTPs, and 1 μL of DNA isolated from the compost samples. Amplification conditions were as follows: initial denaturation at 95°C for 2 min; 35 cycles of denaturation at 95°C for 15 s, annealing at 55°C for 15 s, and extension at 72°C for 30 s; and a final extension step at 72°C for 5 min. PCR products were cloned into T-Vector pMD19 (Takara Bio). The ligation products were used to transform *Escherichia coli* Competent Quick DH5α cells (TOYOBO, Osaka, Japan). Clones were PCR-amplified using primers M13 forward (5′-GTTTTCCCAGTCACGACGTT-3′) and M13 reverse (5′-GGAAACAGCTATGACCATGA-3′) according to standard procedures, and purified with ExoSAP-IT PCR cleanup reagent (USB, Cleveland, OH, USA) according to the manufacturer's instructions. The purified products were sequenced using the BigDye Terminator Cycle Sequencing kit V.3.1 (Applied Biosystems, Carlsbad, CA, USA) according to the manufacturer's instructions. The M13 forward and reverse primers were used for sequencing the plasmids containing the *soxB* fragment. The obtained products were analyzed using the ABI PRISM 3130 Genetic Analyzer (Applied Biosystems).

Phylogenetic analyses and sequence population diversity

The entire *soxB* sequence data, excluding primer sequences and stop codons were used for analysis. Chimeric sequences were omitted using chimera check (FunGene) (1). Headd and Engel (2013) defined the operational taxonomic unit (OTU) criteria as 80%

soxB sequence similarity. In this study, DNA sequences with similarities >80% were considered to represent the same OTU using the mothur software (4). One representative clone from each OTU was randomly selected and homology searches were performed using the BLAST program against the GenBank/EMBL/DDBJ sequence databases. Amino acid sequences of the representative clones were aligned with reference amino acid sequences obtained from the GenBank/EMBL/DDBJ databases using CLUSTAL W (6). Minimum evolution analysis was performed using the MEGA software package, version 5.2.2 (5); the tree topology confidence limits were estimated using 1,000 bootstrap replicates (2). Coverage, and Shannon diversity indices were calculated using the mothur software.

References for Supplementary Information

1) Edgar, R.C., B.J. Haas, J.C. Cleente, C. Quince, and R. Knight. 2011. UCHIME improves sensitivity and speed of chimera detection. Bioinformatics. 27:2194–2200.

2) Felsenstein, J. 1985. Confidence limits on phylogenies: An approach using the bootstrap. Evolution. 39:783–791.

3) Headd, B., and A.S. Engel. 2013. Evidence for niche partitioning revealed by the distribution of sulfur oxidation genes collected from areas of a terrestrial sulfidic spring with differing geochemical conditions. Appl. Environ. Microbiol. 79:1171–1182.

4) Schloss, P.D., S.L. Westcott, T. Ryabin, et al. 2009. Introducing mothur: open-source, platform-independent, community-supported software for describing and comparing microbial communities. Appl. Environ. Microbiol. 75:7537–7541.

5) Tamura, K., D. Peterson, N. Peterson, G. Stecher, M. Nei, and S. Kumar. 2011. MEGA5: Molecular evolutionary genetics analysis using maximum likelihood, evolutionary distance, and maximum parsimony methods. Mol. Biol. Evol. 28:2731–2739.

6) Thompson, J.D., D.G. Higgins, and T.J. Gibson. 1994. CLUSTAL W: improving the sensitivity of progressive multiple sequence alignment through sequence weighting, position-specific gap penalties and weight matrix choice. Nucleic Acids Res. 22:4673–4680.

7) Tourna, M., P. Maclean, L. Condron, M. O'Callaghan, and S.A. Wakelin. 2014. Links between sulphur oxidation and sulphur-oxidizing bacteria abundance and diversity in soil microcosms based on *soxB* functional gene analysis. FEMS Microbiol. Ecol. 88:538–549.

Supplementary Figure Legend

Fig. S1. Temporal changes in temperature during the composting process.

Fig. S2. Relative abundance of phylogenetic groups of *soxB* sequences throughout the composting process. The total numbers of *soxB* sequences were 49 clones (day 1), 61 clones (day 4), 40 clones (day38), 68 clones (day64), and 42 clones (day 106).

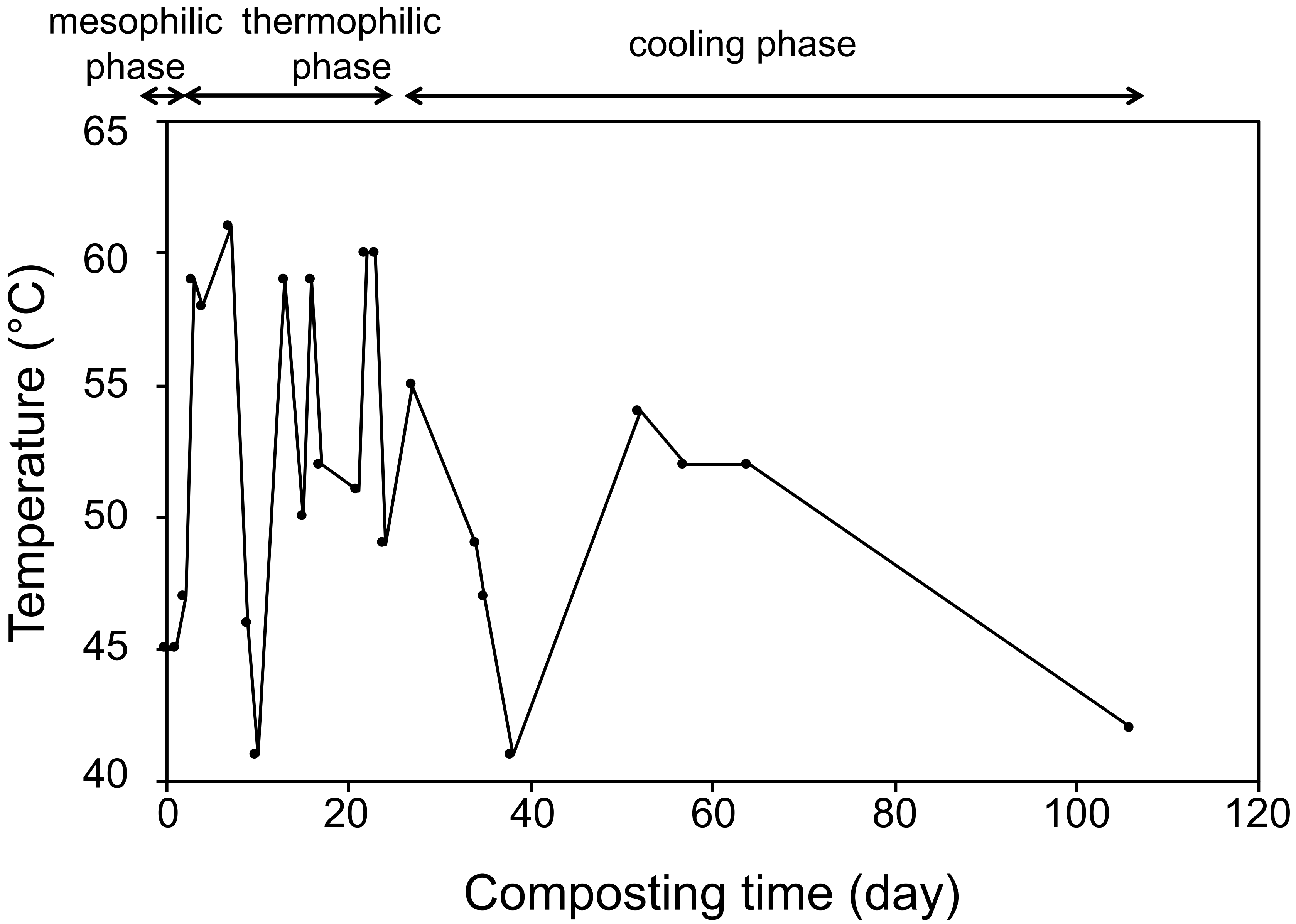

Fig. S1 (Mori et al.)

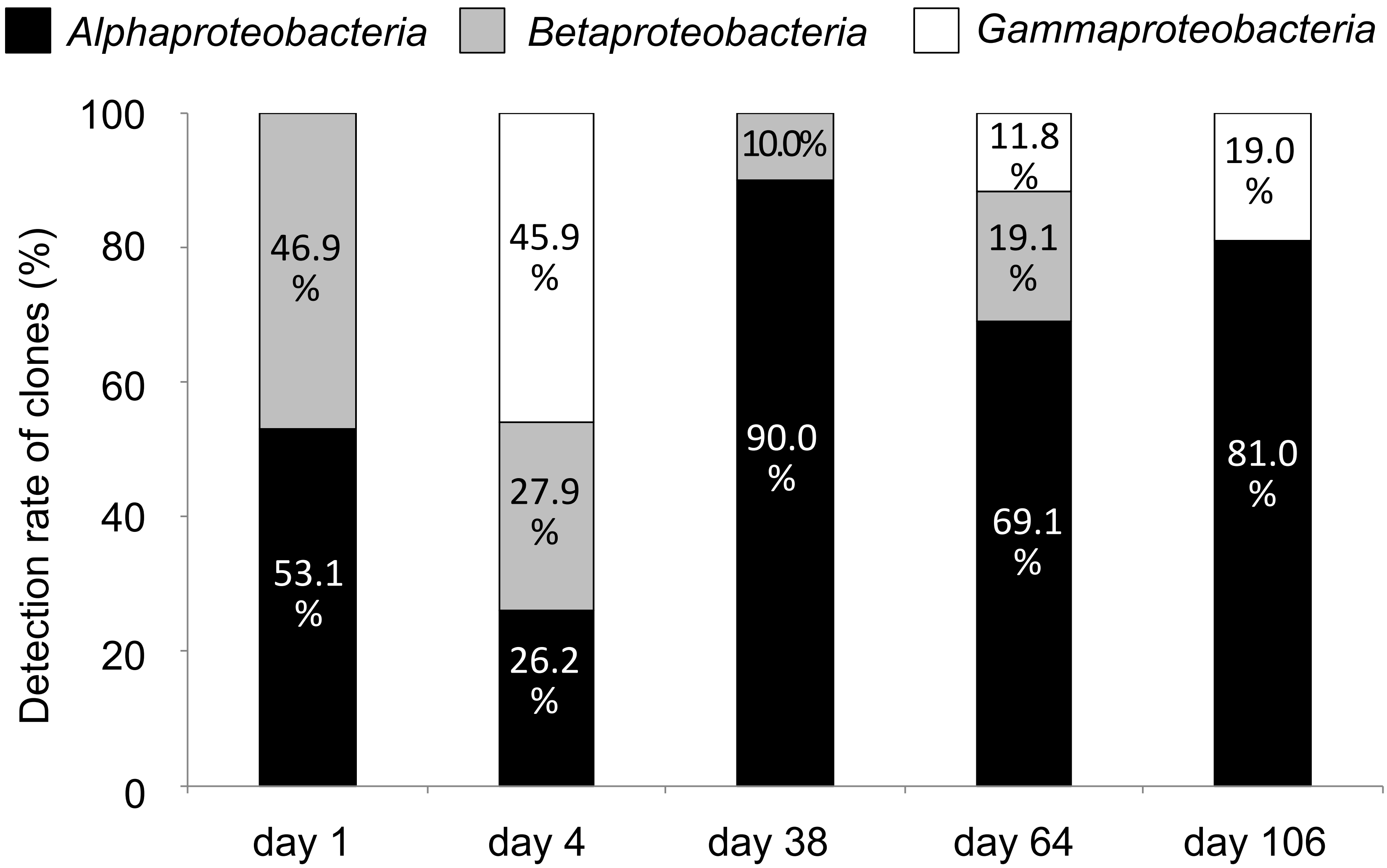

Fig. S2 (Mori et al.)

Table S1. SoxB gene sequence information for investigated compost sequences

OTU	Most similar *soxB* sequence in NCBI based on BLAST search	Accession number	Identity	Isolation source of most related clones	Number of clones				
					day 1	day 4	day 38	day 64	day 106
1	Uncultured bacterium isolate DGGE gel band Sox-9	HQ200289.1	90%	sulfide-rich wastewater of print and dye wastewater treatment plant	10	0	0	0	0
2	Uncultured bacterium clone JFS11	HM209807.1	84%	sulfide-rich wastewater of print and dye wastewater treatment plant	1	0	0	0	0
3	Uncultured bacterium clone 30S1D6_21	KC333027.1	78%	farm soil	1	0	3	8	0
4	*Hydrogenophaga* sp. BB11	KC295220.1	92%	-	9	17	0	0	0
5	Uncultured bacterium clone 16S1D1_17	KC332949.1	77%	farm soil	1	0	0	0	0
6	Uncultured bacterium clone 30S1D1_4	KC332982.1	79%	farm soil	0	0	1	1	0
7	Uncultured bacterium clone SC1	KF789367.1	78%	agricultural soil	0	0	0	4	0
8	Uncultured bacterium clone BROK_0710_012A_22	JX471151.1	79%	terrestrial sulfidic spring, +14 m from orifice	1	0	0	0	0
9	*Azospirillum thiophilum*	JN015012.1	79%	bacterial mat	15	9	7	21	0
10	Uncultured bacterium clone BROK_1010_06B_81	JX471438.1	81%	terrestrial sulfidic spring, +5.5 m from orifice	0	0	0	1	0

11	*Azospirillum thiophilum*	JN015012.1	79%	bacterial mat	1	0	0	3	0
12	Uncultured bacterium clone S2	KU603335.1	78%	production water of Shengli oil field	0	0	1	0	0
13	Uncultured bacterium clone 30S1D1_24	KC333002.1	76%	farm soil	0	1	0	0	0
14	*Hydrogenophaga* sp. RAC07	CP016449.1	80%	Chrysochromulina tobin phycosphere	0	27	0	8	8
15	*Roseobacter* sp. NP30	EU196349.1	83%	cold saline (7.5% salt) sulfidic spring, Canadian High Arctic	1	0	0	0	0
16	Uncultured bacterium clone S10	KU603343.1	80%	production water of Daqing oil field	0	0	1	0	0
17	Uncultured bacterium clone S4	KU603337.1	93%	production water of Xinjiang oil field	1	5	0	0	0
18	Uncultured bacterium clone GHL1_S_soxB_09	JX564917.1	82%	Qinghai-Tibetan lakes	0	1	0	0	0
19	Uncultured bacterium clone SB52	KF789283.1	74%	high saline soil	3	0	3	0	0
20	Uncultured bacterium clone JSS107	JQ256901.1	77%	tidal surface sediment, Janssand	0	1	5	7	14
21	*Mesorhizobium* sp. B7	CP018171.1	79%	Deep seawater in South China Sea	1	0	0	0	1
22	Uncultured bacterium clone MCSoxBD01	FJ604826.1	76%	cave water	0	0	1	0	0

23	Uncultured bacterium clone GHL1_S_soxB_04	JX564914.1	78%	Qinghai-Tibetan lakes	0	0	1	0	0
24	Uncultured bacterium clone CIBA-X26	EU855122.1	72%	coastal aquaculture soil	1	0	11	4	4
25	Uncultured bacterium clone SC143	KF789457	77%	agricultural soil	1	0	1	1	1
26	*Starkeya novella*	AF139113.2	76%	soil	2	0	4	6	8
27	Uncultured bacterium clone JSS041	JQ256873.1	75%	tidal surface sediment, Janssand	0	0	1	4	3
28	Uncultured bacterium clone 16S1D1_7	KC332941.1	82%	farm soil	0	0	0	0	1
29	Uncultured bacterium clone 30S1D1_27	KC333005.1	85%	farm soil	0	0	0	0	1
30	Uncultured bacterium clone 16S1D1_12	KC332945.1	80%	farm soil	0	0	0	0	1

Table S2. Diversity and predicted richness of *soxB* fragments estimated based on Shannon Diversity indices computed using mothur

Library	Number of clones sequenced	Number of OTUs detected	Coverage (%)	Shannon index (Confidence interval)
day 1	49	15	79.6	2.06 (1.76, 2.35)
day 4	61	7	95.1	1.41 (1.20, 1.62)
day 38	40	13	82.5	2.12 (1.85, 2.39)
day 64	68	12	95.6	2.15 (1.95, 2.35)
day 106	42	10	88.1	1.86 (1.58, 2.13)
